# Dose-dependent tissue tropism and efficacy of early BKI-1748 treatment in chronic *Toxoplasma gondii* infection in sheep

**DOI:** 10.1016/j.fawpar.2025.e00297

**Published:** 2025-10-26

**Authors:** Roberto Sánchez-Sánchez, Rafael Calero-Bernal, Natalia Velasco-Jiménez, Irene Gallego-Moreno, Carmen Pérez-Díaz, Rocío Bustamante, Ryan Choi, Matthew A. Hulverson, Andrew Hemphill, Wesley C. Van Voorhis, Luis Miguel Ortega-Mora

**Affiliations:** a SALUVET, Animal Health Department, Faculty of Veterinary Sciences, Complutense University of Madrid, Ciudad Universitaria s/n, 28040 Madrid, Spain; b Animal Medicine and Surgery Department, Faculty of Veterinary Sciences, Complutense University of Madrid, Ciudad Universitaria s/n, 28040 Madrid, Spain; c Center for Emerging and Re-emerging Infectious Diseases (CERID), Division of Allergy and Infectious Diseases, Department of Medicine, University of Washington, 98109-4766, Seattle, Washington, USA; d Institute of Parasitology, Vetsuisse Faculty, University of Bern, Länggass-Strasse 122, CH-3012 Berne, Switzerland

**Keywords:** *Toxoplasma gondii*, Sheep, Chronic infection, Tissue tropism, Food safety, BKI-1748

## Abstract

The presence of microscopic cysts of the zoonotic apicomplexan parasite *Toxoplasma gondii* in mutton is relatively common. *Toxoplasma gondii* is frequently transmitted to humans through the consumption of raw or undercooked meat and infected people may suffer from neurological, ocular and pregnancy disorders. Experimental infections in sheep have provided clues on the *T. gondii* tissue tropism during the chronic stage of infection. However, data regarding infections involving low challenge doses is lacking. Following challenge of sheep with 1000 sporulated oocysts of the Type II TgShSp1 strain, parasite DNA was detected in all sheep at 62 days post-challenge, with detection rates of 87 %, 79 %, 66 % and 66 % in the brain, heart, tongue and *biceps femoris* muscle, respectively. By contrast, after challenge of sheep with a dose of 10 oocysts, parasite DNA was detected in tissues of only 5 out of 8 animals (62.5 %). The *biceps femoris* muscle was the most frequently infected tissue (parasite DNA detection rate of 50 %), resembling the pattern observed in naturally infected sheep. In addition, the administration of multiple doses of the compound BKI-1748, which reached therapeutic concentrations in plasma and cerebrospinal fluid, to infected sheep at 2 and 7 days post-challenge prevented the establishment of the chronic *T. gondii* infection in the treated animals. Therefore, BKI-1748 could be a promising tool for improving safety in mutton intended for human consumption.

## Highlights

• Tissue cyst distribution of *T. gondii* in sheep depends on challenge dose used.
• Infection with 10 oocysts results in zoites detected in edible sheep tissues.
• Early BKI-1748 treatment prevents the establishment of chronic infection in sheep.

## 1 Introduction

*Toxoplasma gondii* is a widespread zoonotic apicomplexan parasite, capable of infecting any **warm-**blooded animal. In veterinary medicine, *T. gondii* is recognized as one of the most relevant infectious agents causing foetal loss and stillbirths in sheep and goats following primary infection through the intake of sporulated oocysts during pregnancy. In humans, congenital infections by *T. gondii* are very relevant due to neurological damage and visual impairment in newborns, while in immunocompromised patients, reactivation of latent infections might lead to acute cerebral or systemic disease (Elsheikha et al., 2020; Bollani et al., 2022). *Toxoplasma gondii* ranks fourth among foodborne zoonotic parasites worldwide (World Health Organization, 2015) and second in Europe (European Food Safety Authority Panel et al., 2018). The main sources of human infection are fresh produce contaminated with oocysts or meat containing bradyzoites (Almeria and Dubey, 2021). Specifically, the consumption of raw or undercooked mutton has been identified as major source of *T. gondii* infection in humans, being estimated to cause over 40 % of foodborne toxoplasmosis in the Eastern Mediterranean countries, although this varies depending on culinary habits and environmental factors (Hoffmann et al., 2017).

In a systematic review of *T. gondii* detection by direct methods in mutton worldwide, the pooled prevalence was 14.7 %, although a high heterogeneity of the studies and prevalence rates in natural infections were reported (Belluco et al., 2016). The dynamics of experimental *T. gondii* infection have been comprehensively studied in sheep. During early stages of infection, the tachyzoites replicate in the small intestine and mesenteric lymph nodes before disseminating systemically *via* the bloodstream (Sánchez-Sánchez et al., 2025a). From day 14 post-infection (pi), due to the influence of the immune response, tachyzoites transform into bradyzoites and form tissue cysts (chronic stage), which are mainly confined in the brain and striated muscles, where they may remain viable lifelong. Several studies have examined chronic infections under experimental conditions in sheep, highlighting the influence of the infectious dose of oocysts in the detection frequency and distribution of mature tissue cysts (Dubey, 1984; Lundén and Uggla, 1992; Katzer et al., 2014; Juránková et al., 2015; Thomas et al., 2022). Studies employing a low oocyst dose (100 oocysts or fewer) for challenging sheep, potentially more accurate in mimicking natural oocyst-driven infections, are scarce and only few studies evaluated the chronic phase after infection with different oocyst doses (dose titration) (Esteban-Redondo et al., 1999).

Effective control of *T. gondii* infections in humans and animals requires One Health integrative efforts (Aguirre et al., 2019). From a food safety point of view and aiming to mitigate meat-borne toxoplasmosis in humans, current meat sanitary inspection tools are not capable of detecting microscopic *T. gondii* tissue cysts in slaughtered animals. Therefore, current preventive measures are targeted towards the proper inactivation of tissue cysts, mainly through freezing and cooking of the meat (Kuruca et al., 2023). The only available live attenuated vaccine was designed to prevent abortion in sheep (reviewed by Dubey, 2009), but its capacity to reduce the tissue cyst burden was also demonstrated (Katzer et al., 2014). In sheep, classical drugs tested for protection against congenital toxoplasmosis yielded poor protection rates even when administered prophylactically, and therefore new, safe and more effective compounds are needed (Sanchez-Sanchez et al., 2018). Several molecules with activity against the chronic stage (bradyzoites within tissue cysts) have been described *in vitro*, however, *in vivo* efficacy has been scarcely studied (Montazeri et al., 2018). In lambs, toltrazuril applied from the second week pi onwards, exhibited a partial reduction of tissue cysts (Kul et al., 2013). In recent years, Bumped Kinase Inhibitors (BKIs), that target calcium-dependent protein kinase 1 (CDPK1) and mitogen-activated protein kinase-like 1 (MAPKL1), emerged as a promising class of drugs for a safer and more effective treatment of congenital toxoplasmosis (Ojo et al., 2010; Van Voorhis et al., 2021; Montgomery et al., 2025). BKI-1553 (compound 32) was shown to be effective against latent toxoplasmosis if applied in mice five weeks pi (Vidadala et al., 2016). The lead compound BKI-1748 has a good safety profile, with low affinity for human ether-à-go-go related gene (cardiovascular safety in humans) and acceptable cardiovascular safety testing in rat and dog, *in vitro* efficacy against *T. gondii* with 50 % effective concentration (EC50) in the nanomolar range and high efficacy in non-pregnant and pregnant mouse models of toxoplasmosis (Hulverson et al., 2019; Imhof et al., 2021). In pregnant sheep, BKI-1748 was found be safe and effective in reducing abortions and lamb mortality in the early neonatal period, as well as preventing congenital infection when administered from the first week pi (Sánchez-Sánchez et al., 2024, Sánchez-Sánchez et al., 2025b).

In this context, the present study aims at providing further understanding of 1) the tissue tropism of chronic *T. gondii* infection in sheep infected experimentally with high and low doses of type II *T. gondii* oocysts; 2) the ability of BKI-1748 to reach therapeutic concentrations against *T. gondii* zoites in the cerebrospinal fluid (CSF) and 3) the efficacy of the compound on the establishment of the chronic infection in sheep treated from days 2 or 7 pi onwards.

## 2 Materials and methods

### 2.1 Ethics statement

The experiment was authorized by the Animal Welfare Committee of the Community of Madrid, Spain (PROEX 064/19, 128.1/21 and 210.0/22), following procedures described in Spanish and European Union legal requirements (Law 32/2007, R.D. 53/2013, and Council Directive 2010/63/EU). Good clinical practices were implemented to ensure animal welfare.

### 2.2 Animals and experimental design

**Fig. 1:**
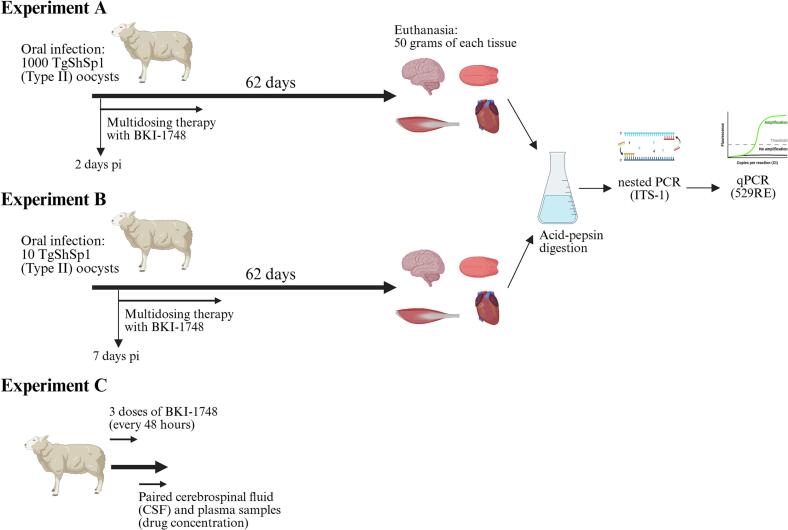
**Experimental design.** Adult sheep from experiments A and B were challenged at 90 days of pregnancy. The oocysts were obtained after faecal flotation (purification) of faeces produced by a kitten that had been fed mice chronically infected with the TgShSp1 isolate (Sánchez-Sánchez et al., 2019). Oocysts were left to sporulate for one week in 2 % H_2_SO_4_ and then stored for 13 months at 4 °C before challenge. The TgShSp1 isolate had demonstrated cystogenic capability according to previous studies *in vitro* and in mice (Colos-Arango et al., 2023). BKI-1748 treatment was applied orally at 15 mg/kg every 48 h, with 10 doses in experiments A and B and only 3 doses in experiment C. This figure was created with https://BioRender.com.

Tissue samples from dams were collected in studies that evaluated the efficacy of BKI-1748 in reducing foetal mortality and congenital infection in *T. gondii* experimentally infected sheep (Sánchez-Sánchez et al., 2024, Sánchez-Sánchez et al., 2025b). In both studies, 18-month-old sheep were infected with *T. gondii* sporulated oocysts of the *T. gondii* isolate TgShSp1 (ToxoDB genotype #3) (Sánchez-Sánchez et al., 2019) at 90 days of pregnancy and monitored for two months until delivery. In experiment A (Sánchez-Sánchez et al., 2024), 18 pregnant sheep were divided into three experimental groups: G1a (*n* = 7) and G2a (*n* = 8) were infected with 1000 sporulated oocysts, while G3a (*n* = 3) served as the uninfected control group. In experiment B (Sánchez-Sánchez et al., 2025b), 19 pregnant sheep were divided into three experimental groups: G1b (n = 8) and G2b (n = 8) were infected with 10 sporulated oocysts, while sheep in G3b (n = 3) were mock dosed (uninfected group). BKI-1748 treatment was administered orally in 10 doses at 15 mg/kg every 48 h, starting on day 2 pi for G1a and on day 7 pi for G1b (Fig. 1).

### 2.3 Sample collection and acid-pepsin digestion

At 62 days pi, experimental animals of all groups were sedated with 0.1 mg/kg of xylazine by the intravenous route (Rompun, Bayer, Mannheim, Germany) and then immediately euthanized by an intravenous overdose of embutramide and mebezonium iodide (T61, Intervet, Salamanca, Spain). During necropsy 50 g of brain, apex of the heart, *biceps femoral* muscle and the tip of the tongue were collected and stored at −20 °C until analysis. Muscle tissues (heart, *biceps femoral* muscle and tongue) were subjected to acid-pepsin artificial digestion as previously described (Dubey, 2010). As lower efficiency of acid-pepsin digestion is expected for brain tissues (Dubey, 1998), these were homogenized in PBS, filtered through sterile gauze and centrifuged at 1350 ×*g* for 15 min at 4 °C (Largo-De la Torre et al., 2022). The extracts from muscle tissues and brain homogenates were weighed and stored at −80 °C until DNA extraction.

### 2.4 DNA extraction and PCR for parasite detection and quantification

Extracts from muscle tissues and brain homogenates were centrifuged at 3500 ×*g* for 10 min at 4 °C and supernatants were removed. Genomic DNA extraction from three aliquots of 50–100 mg of each extract from muscle tissues and brain homogenate was carried out using the commercial Maxwell RSC Tissue DNA Kit (Promega, Wisconsin, USA) following the manufacturer's recommendations. The DNA concentration of each sample was determined using a Synergy® H1 multimode microplate reader (Biotek, Winooski, USA) and Gen5 version 2.09.1 software (Biotek, Winooski, USA). After adjustment to 100 ng/μL, *T. gondii* DNA detection was carried out by nested PCR (nPCR) of the ITS-1 region (Hurtado et al., 2001). Each reaction was performed in a final volume of 25 μL with 5 μL of sample DNA. Positive *T. gondii* samples equivalent to 10 (*n* = 2), 1 (*n* = 3), and 0.1 (n = 2) tachyzoites in 100 ng of sheep genomic DNA were included in each batch of amplifications. Ten-μL aliquots of the nPCR products were visualized under UV light in 1.8 % agarose gel stained with GelRed® nucleic acid gel stain (Biotium, Inc., Fremont, CA, USA). A reaction was determined as positive when a band of 227 bp was observed.

Additionally, DNA resulting as positive by nPCR were diluted to 20 ng/μL and quantified using qPCR as previously described (Gutierrez et al., 2012) with minor modifications. Primer pairs for the 529RE fragment and for the β-actin gene were used to quantify the parasite and host specific DNA, respectively. Reactions were performed in a final volume of 20 μL using Go Taq® qPCR Master Mix (Promega, Wisconsin, USA), 20 pmol of each primer, and 100 ng of DNA in the ABI 7500 Fast Real-time PCR System (Applied Biosystems, Foster City, CA, USA). Amplification was performed by a standard protocol (10 min at 95 °C, 40 cycles at 95 °C for 15 s, and 60 °C for 1 min). The number of *T. gondii* tachyzoites was calculated by interpolating the Ct values on two standard curves: 1) one curve equivalent to 10^5^ to 10^−1^ tachyzoites with 10-fold serial dilutions in a solution of ovine genomic DNA, and 2) a curve of 320, 160, 80, 40, 20, 10, and 5 ng of genomic DNA for ovine DNA quantification. Standard curves for *T. gondii* and sheep DNA showed an average slope of −3.38 and − 3.34, respectively, and a R^2^ > 0.99.

### 2.5 Assessment of the drug levels and protein binding of BKI-1748 in the cerebrospinal fluid

In experiment C, six non-pregnant, three-year-old Rasa Aragonesa sheep were orally dosed with three doses of BKI-1748 at 15 mg/kg every 48 h. The compound was dissolved in a vehicle containing 60 % PHOSAL 53 MCT, 30 % PEG 400 %, and 10 % ethanol as previously described (Sánchez-Sánchez et al., 2024, Sánchez-Sánchez et al., 2025b). Paired samples of blood and cerebrospinal fluid (CSF) were collected just before or after the 3rd dose, with the following schedule: pre-dose and 36 h after dosing (two sheep); 12 and 60 h after dosing (two sheep); and 24 and 72 h after dosing (two sheep) (Fig. 1).

Blood was collected *via* jugular venipuncture and placed in 1 mL heparin tubes to obtain plasma for pharmacokinetics (PK) analysis after centrifugation. Sheep were sedated with intravenous diazepam (0.4 mg/kg; Ziapam, Ecuphar, Barcelona, Spain), followed by induction with a mixture of ketamine (2 mg/kg; Anesketin, Dechra, León, Spain) and propofol (2 mg/kg; Vetofol, 10 mg/mL, Norbrook Laboratories, Monaghan, Ireland) administered intravenously. Following orotracheal intubation, general anaesthesia was maintained with isoflurane (1.5–2 %; Isofluorin, Fatro Ibérica, Barcelona, Spain) vaporized in 100 % oxygen and delivered through a circle breathing system. Physiological parameters were monitored throughout the procedure. The CSF was collected *via* puncture of the cerebellomedullary cistern using a spinal needle (22G x 63 mm, reference 405244, Becton Dickinson, Franklin Lakes, NJ, USA) following the protocol described by Ramos-Antón and Ferrer-Mayayo (2007). CSF was collected by dripping into 0.5 mL polypropylene tubes for PK analysis. Plasma and CSF samples were stored at −20 °C until PK analysis.

BKI-1748 concentrations in plasma and CSF were determined as previously described (Hulverson et al., 2019). Briefly, BKI-1748 was extracted from plasma or CSF samples using a volume of 95 μL of 80:20 acetonitrile:water with an internal standard added to a 5 μL sample of plasma or CSF and mixed for 30 min. The mixture was centrifuged at 4000 x*g* for 10 mins. Supernatant was removed and quantified by liquid chromatography-mass spectrometry/mass spectrometry analysis with a Acquity ultra performance liquid chromatography system in tandem with a Xevo TQ-S mass spectrometer (Waters, Milford, MA). A matrix-matched standard curve was prepared for comparison and quantification.

The degree of protein binding in CSF was determined by equilibrium dialysis (HTD 96B, HTDialysis, Connecticut, USA). BKI-1748 was added to 120 μL of CSF at a concentration of 5 μM. 20 μL of this sample was reserved as a 100 % recovery standard and the remaining volume was added to the donor side of the dialysis wells (3.5 kDa MW cutoff, HTDialysis catalog no. 1135). 100 μL of dialysis buffer (100 mM Na_2_HPO_4_, 150 mM NaCl, pH 7.4) was added to the acceptor side and wells were sealed and incubated for 16 h at 37 °C on an orbital shaker at 100 rpm. After incubation, 20 and 80 μL aliquots were obtained from the donor and acceptor sides, respectively, and matrix adjusted with CSF or buffer. BKI-1748 was extracted using 80:20 acetonitrile:water with an internal standard. Samples were analysed on an Acquity ultra-performance liquid chromatography (UPLC) system in tandem with a Xevo TQ-S micro mass spectrometer (Waters, Massachusetts, USA). Fraction of compound bound to protein was calculated as bound/(unbound+bound) while accounting for dilutions for matrix adjustments.

### 2.6 Statistical analysis

For the infected/untreated groups (G2a and G2b), differences in parasite DNA detection rates and burdens in different tissues and within tissues were evaluated using Fisher's exact test (for parasite DNA detection rates) and the non-parametric Kruskal–Wallis test followed by Dunn's and Mann–Whitney tests (for parasite burdens). Statistical significance for all analyses was established at *P* < 0.05. All statistical analyses were performed using GraphPad Prism 8.0.1 software (San Diego, CA, USA).

## 3 Results

### 3.1 Detection of *T. gondii* DNA in tissues of chronically infected sheep

As shown in the original publications (Sánchez-Sánchez et al., 2024, Sánchez-Sánchez et al., 2025b), all challenged animals (G2a and G2b) seroconverted (IgG) at 14–21 pi and thus were considered infected and suitable for further tropism studies at the chronic stage of the infection. The results of the parasite DNA detection are summarized in Table 1. In experiment A, in the animals challenged with 1000 oocysts (group G2a), parasite DNA was detected in all sheep (8/8) and in at least one replicate of each analysed tissue. Parasite DNA detection rates were 87.5 % (21/24), 79.2 % (19/24), 66.7 % (16/24) and 66.7 % (16/24) in the brain, heart, tongue and *biceps femoris* muscle, respectively, without statistically significant differences between them. In experiment B, in the group challenged with 10 oocysts (group G2b), parasite DNA was detected in 5 out of 8 infected sheep (62.5 %), with 3 of them positive in two tissues (the *biceps femoris* muscle and either the tongue or the heart), and 2 of them positive only in the *biceps femoris* muscle. Parasite DNA detection rates were 50 % (12/24) in the *biceps femoris* muscle, 20.8 % (5/24) in the heart, 12.5 % (3/24) in the tongue and 0 % (0/24) in the brain. Significantly higher DNA detection rates were observed in the *biceps femoris* muscle and heart compared to brain (*biceps femoris* muscle *vs* brain, *p* < 0.0001; heart *vs* brain, *p* < 0.05). Furthermore, the parasite DNA detection in the *biceps femoris* muscle was significantly higher compared to tongue (p < 0.05). Parasite DNA was not detected in tissues collected from uninfected groups (G3a and G3b).

**Table 1:** *Toxoplasma gondii* DNA detection in tissues collected in the chronic phase of sheep experimentally infected with either 1000 or 10 TgShSp1 sporulated oocysts.

**Group**	**Challenge dose**	**Sheep identification**	**Tissues**			
			**Brain**	**Heart**	***Biceps femoris* muscle**	**Tongue**
**G2a**	**1000 TgShSp1 sporulated oocysts**	1a	3/3	1/3	2/3	1/3
		2a	2/3	3/3	2/3	2/3
		3a	3/3	3/3	3/3	2/3
		4a	1/3	2/3	1/3	1/3
		5a	3/3	2/3	1/3	3/3
		6a	3/3	3/3	2/3	2/3
		7a	3/3	3/3	3/3	2/3
		8a	3/3	2/3	2/3	3/3
		Detection rate⁎	21/24 (87.5 %)	19/24 (79.2 %)	16/24 (66.7 %)	16/24 (66.7 %)
**G2b**	**10 TgShSp1 sporulated oocysts**	1b	0/3	0/3	3/3	3/3
		2b	0/3	0/3	2/3	0/3
		3b	0/3	0/3	0/3	0/3
		4b	0/3	0/3	0/3	0/3
		5b	0/3	3/3	3/3	0/3
		6b	0/3	2/3	1/3	0/3
		7b	0/3	0/3	0/3	0/3
		8b	0/3	0/3	3/3	0/3
		Detection rate⁎	0/24 (0 %)^b^	5/24 (20.8 %)^a^	12/24 (50 %)^a, c^	3/24 (12.5 %)^d^

⁎ For parasite DNA detection rate, the number of positive replicates among the three replicates analysed per tissue; percentages are shown in brackets. Different letters (a or b and c or d) mean statistically significant differences between tissues in Experiment B. Biceps femoris muscle *vs* brain, p < 0.0001; Biceps femoris muscle *vs* tongue (p < 0.05); Heart *vs* brain, p < 0.05.

Comparing each tissue among the two experimentally infected groups, higher parasite DNA detection rates were observed in the brain (p < 0.0001), heart (*p* < 0.001), and tongue (p < 0.001) of sheep challenged with 1000 oocysts (G2a) than in sheep challenged with 10 oocysts (G2b), whereas no significant differences between both groups were observed in the parasite DNA detection rates in the *biceps femoris* muscle (Table 1).

### 3.2 Burden of *T. gondii* zoites in tissues of the chronically infected sheep

**Fig. 2:**
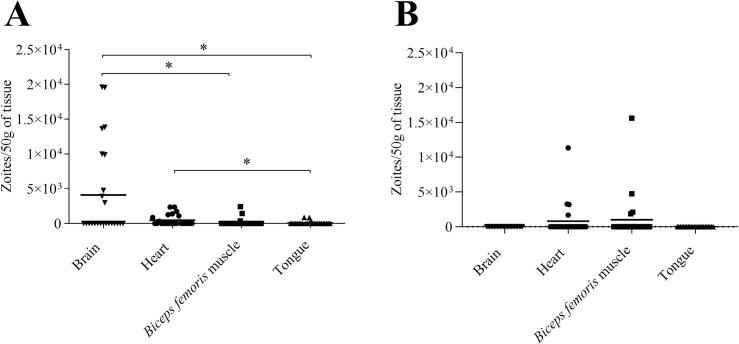
**Dot-plot graphs of the *Toxoplasma gondii* burden in sheep tissues collected at the chronic phase of infection.** In A, sheep were dosed with 1000 oocysts and in B, sheep were dosed with 10 oocysts. Each dot represents an individual value of the estimated parasite burden, and the means are represented as horizontal lines. Negative samples were represented as 0 parasites. Parasite burdens were analysed *via* the nonparametric Kruskal–Wallis test followed by Dunn's test for comparisons between different tissues, as well as the Mann–Whitney test for pairwise comparisons. For significant differences between tissues, (*) indicates *p* < 0.05.

In the tissues collected from sheep infected with 1000 oocysts (G2a), 23 out of 72 (32 %) samples that resulted positive by nPCR were quantified. A higher parasite burden was observed in the brain (mean 4088; range: 0–19,612 zoites/50 g of tissue) compared to *biceps femoris* muscle (mean 176; range: 0–1444 zoites/50 g of tissue) (p < 0.05) and tongue (mean 89; range: 0–860 zoites/50 g of tissue) (p < 0.05). Additionally, a higher number of parasites was observed in the heart (mean 345; range: 0–2362 zoites/50 g of tissue) compared to tongue (p < 0.05) (Fig. 2A).

In the tissues collected from sheep infected with 10 oocysts (G2b), 8 out of 20 (40 %) samples that had tested positive by nPCR were quantified, belonging to *biceps femoris* muscle (mean 1013; range: 0–15,617 zoites/50 g of tissue) and heart (mean 812; range 0–11,352 zoites/50 g of tissue). No significant differences in the parasite burden were observed between tissues (Fig. 2B).

Comparing each tissue among the two experimentally infected groups, a higher cerebral parasite burden was detected in sheep challenged with 1000 oocysts (G2a) compared to 10 oocysts (G2b) (*p* < 0.01), while no significant differences between challenge doses were observed in heart, *biceps femoris* muscle, and tongue.

### 3.3 Pharmacokinetics of BKI-1748 in sheep cerebrospinal fluid

All CSF samples could be collected except for one sheep at 36 h after dosing. The CSF samples collected from one sheep at 12 and 60 h after dosing appeared highly viscous upon macroscopic examination and were therefore discarded.

**Fig. 3:**
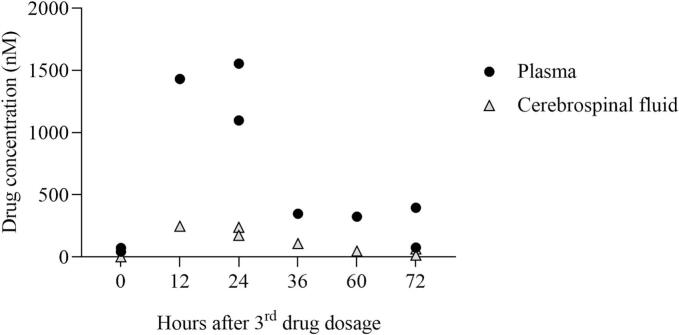
**Dot-plot graph of BKI-1748 exposure in plasma and cerebrospinal fluid (CSF) of dosed sheep.** Each point represents individual data of drug exposure. Sheep were dosed orally with 15 mg/kg of BKI-1748 three times at 48-h intervals, and samples were collected after the third dose.

Maximum BKI-1748 plasma concentrations of 1431–1554 nM were reached in sheep sampled at 12–24 h after administration of the third dose with concentrations of 75–96 nM found at 72 h post-dosing. A similar trend to that observed in plasma BKI-1748 levels was observed in the CSF, with maximum concentrations of 239–246 nM at 12–24 h after the third dose was administered, and lower levels (14–64 nM) at 72 h post-dosing (Fig. 3). CSF-to-plasma ratio for the compound was 0.15–0.19 at 12, 24, 60 and 72 h after dosing and 0.31 at 36 h after dosing (Supplementary file 1). Ultimately, 9.5 % of BKI-1748 was bound to sheep CSF proteins.

### 3.4 Full protection of BKI-1748 treatment against the establishment of the chronic T. gondii infection in sheep

Both groups of infected and then treated sheep (G1a and G1b) were treated with BKI-1748 following the same dose regime (10 doses of 15 mg/kg every 48 h); for G1a (infected with 1000 oocysts) starting the treatment at day 2 pi and for G1b (infected with 10 oocysts) starting the treatment at day 7 pi. *T. gondii* DNA was not detected by nPCR in any of the tissues (brain, heart, tongue and *biceps femoris* muscle) collected from the treated sheep (groups G1a and G1b).

## 4 Discussion

A number of surveys have been performed worldwide in recent decades, showing relevant serological evidence of *T. gondii* infection (Dubey et al., 2020; Huertas López et al., 2023) and detection of the chronic infection in naturally infected sheep (Belluco et al., 2016). Focusing on the predilection sites, studies addressing the anatomical distribution of the tissue cysts ranked the selected tissues for this study (brain, heart and skeletal muscles) at the top of the infected tissues in sheep (Opsteegh et al., 2016). Also following previous recommendations (European Food Safety Authority Panel et al., 2018; Rani and Pradhan, 2020), we evaluated 50 g of tissue and concentrated the potential zoites present in the sample in order to improve diagnostic sensitivity. Muscle tissues require an aggressive method (acid-peptic digestion) to release cysts. In contrast to digestion in muscle tissue, acid-pepsin digestion is not optimal in the brain because of its high lipid content; therefore, milder methods such as filtration were employed (Dubey, 1998; Puvanesuaran et al., 2012; Opsteegh et al., 2020). On the other hand, this study cannot ascertain the viability of the detected parasites, since mouse bioassays or alternative molecular assessments (eg. RT-qPCR) were not performed (Marín-García et al., 2022).

Although several studies were conducted, only a few of them tested different doses of oocysts in experimental infections (Dubey and Sharma, 1980; Esteban-Redondo and Innes, 1998; Esteban-Redondo et al., 1999). Therefore, the influence of the dose of oocysts on parasite detection and tissue tropism during chronic infection remains understudied. Similar to the results presented herein using 1000 oocysts, most of the previous studies in sheep using high doses of oocysts reported more frequent parasite detection in the brain and heart than in skeletal muscles (Esteban-Redondo and Innes, 1998; Esteban-Redondo et al., 1999). In terms of parasite quantification, similar to our results, one study highlighted the brain as a major predilection site for the parasite, allowing zoites to largely escape cell-mediated immune response (Juránková et al., 2015). A high proportion of sheep experimentally infected with 400 sporulated Type II oocysts were also found to have cysts in their brains (Mévélec et al., 2010; Ducournau et al., 2020). The present study and most of the previous studies addressing chronic infection in experimentally infected sheep tested Type II isolates which are representative of the predominant *T. gondii* lineage in Europe and North America (Galal et al., 2019; Fernández-Escobar et al., 2022). An experimental study of piglets, which used the same isolate (TgShSp1), infection dose (1000 oocysts) and route of infection as in the present study, as well as the same experimental methodology (Largo-De la Torre et al., 2022), detected higher parasite loads in piglet tissues than in sheep tissues, including the brain, tongue, skeletal muscles and heart, the latter exhibiting 10-fold higher parasite loads.

The infectious dose for sheep in natural conditions is highly variable and depends on the quantity of oocysts in water or feed and their loss of infectivity over time. The lower parasite detection rate observed in naturally infected lambs compared to lambs that were experimentally infected with 1000 oocysts (Thomas et al., 2022) supports the hypothesis that most natural infections involve low oocyst numbers (Dubey, 2021a). Notwithstanding, experimental studies in sheep using low doses of oocysts (100 oocysts or fewer) are scarce (Dubey and Sharma, 1980). In this study, comparison of the infections with high and low oocyst doses showed that the parasite DNA was detected in all sheep of the former group and in five out of eight (62 %) sheep in the latter group. However, the absence of *T. gondii* DNA detection in some animals challenged with the low oocyst dose does not rule out the parasite presence in other locations within the analysed tissues or in other organs. Indeed, all sheep challenged with 10 oocysts had exposure to *T. gondii* and seroconverted (Sánchez-Sánchez et al., 2025b), although antibody detection has shown a moderate/low correlation with the presence of *T. gondii* DNA in tissues from naturally infected sheep (Opsteegh et al., 2016) and in animals experimentally infected with low doses of oocysts (Dubey et al., 1996).

When the parasite DNA detection rates were compared between the two groups of infected sheep, lower rates were observed in the brain, heart and tongue of sheep that were challenged with 10 oocysts than those challenged with 1000 oocysts. The lower detection rate at a low infection dose is probably due to the host immune response, which is more efficient at controlling parasite proliferation during the acute phase of infection (Dubey, 2021b). Also, an effective immune response resulting from a low-dose challenge may prevent the parasite crossing the blood-brain barrier (BBB). In the brains from the group infected with the lower dose, parasite DNA was not detected, which is consistent with previous reports in sheep (Dubey and Sharma, 1980) and piglets (Dubey et al., 1996) challenged with low oocyst doses. However, parasite presence in the brain from sheep challenged with a low oocyst dose cannot be ruled out as the cyst burden could be below the detection limit (analytical sensitivity) of the PCR method used. For the *biceps femoris* muscle, no differences in parasite DNA detection or burden were observed between the high and low doses of infection, similar to previous reports (Dubey and Sharma, 1980), although whether this occurs in other skeletal muscles was not assessed in this study. Furthermore, a high parasite load was detected in some muscle samples collected from the low-dose group, possibly due to an uneven cyst distribution in the muscle tissue. The uneven distribution of the cysts in the muscles of infected animals intended for human consumption is supported by studies, which report that more than half of the people who consume raw or undercooked meat from infected animals do not become infected (Dubey, 2021a). In fact, an erratic distribution of tissue cysts within muscles may have led to an overestimation of the risk in quantitative predictive models (Crotta et al., 2017). Experimental infections with other parasites such as *Trichinella spp.* and *Sarcocystis spp.* also found a different muscle tropism of the parasites depending on the infectious dose, which is probably associated with the degree of vascularization (O'Donoghue and Ford, 1984; Serrano et al., 1999).

The early multi-dosing treatment with BKI-1748 resulted in therapeutic plasma concentrations that inhibited tachyzoite proliferation for 20 days (Sánchez-Sánchez et al., 2024, Sánchez-Sánchez et al., 2025b). In order to gain insight into the anti-parasitic efficacy of BKI-1748 in brain tissues, we evaluated the capacity of the compound to cross the BBB. The concentrations of BKI-1748 in sheep CSF (100–200 nM until 36 h after administration) reached levels of approximately 20–30 % of those found in plasma, which is similar to the results observed in mice treated with the close analogue, BKI-1597 (Hulverson et al., 2019). Thirty-six hours after administration, the estimated free BKI-1748 in the central nervous system (CNS) is above the half-maximal effective concentration of the compound against *T. gondii* tachyzoite proliferation *in vitro* (EC50) (Hulverson et al., 2019; Imhof et al., 2021) which might contribute to reduce the establishment of chronic infection and formation of tissue cysts in infected and treated sheep. Although further studies are needed to determine the *in vitro* efficacy of BKI-1748 against bradyzoites, the knowledge of BBB penetration of the compound could help to refine future *in vivo* studies.

At the beginning of BKI-1748 treatment, 2 and 7 days pi (Sánchez-Sánchez et al., 2024, Sánchez-Sánchez et al., 2025b), *T. gondii* tachyzoites were likely replicating in the small intestine or at the onset of systemic spread (Sánchez-Sánchez et al., 2025a). BKI-1748 treatment prevented the establishment of chronic infection in sheep, whereas a previous study in mice testing the compound administered from day 2 pi (Imhof et al., 2021) showed only a mild reduction in parasite DNA detection in the maternal brain probably due to the shorter period of treatment or shorter intestinal transit time of oocysts in mice than in sheep (De Vega et al., 1998; Padmanabhan et al., 2013). The prevention of the establishment of the *T. gondii* chronic infection conferred by BKI-1748 administered from day 2 or 7 pi is on par with the significant reduction in parasite DNA detection during the chronic phase in lambs vaccinated with the attenuated live vaccine (Toxovax™) (Katzer et al., 2014) and with the reduced brain cyst load in sheep immunized with MIC1-MIC knockout tachyzoites (Mévélec et al., 2010). Although the present study did not evaluate the protective effect of the compound beyond day 7 pi, delaying the start of treatment might reduce its efficacy, as previously observed in *T. gondii*-infected lambs in which tissue cyst burdens were mildly reduced after treatment with toltrazuril from day 14 pi onwards (Kul et al., 2013).

## 5 Conclusions

In conclusion, the present findings support the differential *T. gondii* DNA detection and parasite loads in target tissues of chronically infected sheep depending on the challenge dose. They also demonstrate, for the first time, that sheep infected with as few as 10 oocysts harbour tissue cysts in meat intended for human consumption. Furthermore, the absence of *T. gondii* DNA in the tissues of animals treated with BKI-1748 at an early stage of infection suggests that this compound could improve the food safety of sheep meat.

## CRediT authorship contribution statement

**Roberto Sánchez-Sánchez:** Writing – review & editing, Writing – original draft, Methodology, Investigation, Formal analysis, Conceptualization. **Rafael Calero-Bernal:** Writing – review & editing, Methodology, Conceptualization. **Natalia Velasco-Jiménez:** Writing – review & editing, Investigation. **Irene Gallego-Moreno:** Writing – review & editing, Investigation. **Carmen Pérez-Díaz:** Writing – review & editing, Investigation. **Rocío Bustamante:** Writing – review & editing, Investigation. **Ryan Choi:** Writing – review & editing, Investigation. **Matthew A. Hulverson:** Writing – review & editing, Investigation. **Andrew Hemphill:** Writing – review & editing, Funding acquisition. **Wesley C. Van Voorhis:** Writing – review & editing, Project administration, Funding acquisition. **Luis Miguel Ortega-Mora:** Writing – review & editing, Project administration, Funding acquisition, Conceptualization.

## Declaration of competing interest

The authors declare the following financial interests/personal relationships which may be considered as potential competing interests:

Dr. Wesley C. Van Voorhis is the President and co-owner of ParaTheraTech Inc., a company that is developing BKIs for animal health. Dr. Van Voorhis did not perform the experiments or interpret the results of the experiments, but he did edit this paper and helped plan the experiments. The other authors declare that they have no competing interests.

## Appendix A Supplementary data

Supplementary File 1 BKI-1748 levels in plasma and cerebrospinal fluid (CSF) and CSF-to-plasma ratios in experimentally dosed sheep. Supplementary File 1
