# Supplementary material for: Dose-dependent tissue tropism and efficacy of early BKI-1748 treatment in chronic Toxoplasma gondii infection in sheep
Source: Food Waterborne Parasitol. 2025 Oct 26;41:e00297. doi: 10.1016/j.fawpar.2025.e00297 (PMC12642135; doi:10.1016/j.fawpar.2025.e00297)
Supplement: Supplementary File 1 — BKI-1748 levels in plasma and cerebrospinal fluid (CSF) and CSF-to-plasma ratios in experimentally dosed sheep. [file mmc1.docx]

| Hours after dosing |  | Sheep 1 | Sheep 2 | Sheep 3 | Sheep 4 | Sheep 5 |
| --- | --- | --- | --- | --- | --- | --- |
| 0 | CSF (nM) | 0 | 7 |  |  |  |
|  | Plasma (nM) | 43 | 72 |  |  |  |
|  | Ratio* | **0.00** | **0.10** |  |  |  |
| 12 | CSF (nM) |  |  | 247 |  |  |
|  | Plasma (nM) |  |  | 1431 |  |  |
|  | Ratio* |  |  | **0.17** |  |  |
| 24 | CSF (nM) |  |  |  | 240 | 175 |
|  | Plasma (nM) |  |  |  | 1554 | 1097 |
|  | Ratio* |  |  |  | **0.15** | **0.16** |
| 36 | CSF (nM) |  | 108 |  |  |  |
|  | Plasma (nM) |  | 347 |  |  |  |
|  | Ratio* |  | **0.31** |  |  |  |
| 60 | CSF (nM) |  |  | 48 |  |  |
|  | Plasma (nM) |  |  | 324 |  |  |
|  | Ratio* |  |  | **0.15** |  |  |
| 72 | CSF (nM) |  |  |  | 65 | 15 |
|  | Plasma (nM) |  |  |  | 396 | 76 |
|  | Ratio* |  |  |  | **0.16** | **0.19** |

**Supplementary file 1.** BKI-1748 levels in plasma and cerebrospinal fluid (CSF) and CSF-to-plasma ratios in experimentally dosed sheep.

*Ratio = CSF concentration / plasma concentration
